# Interface Friction of Double-Walled Carbon Nanotubes Investigated Using Molecular Dynamics †

**DOI:** 10.3390/mi8030084

**Published:** 2017-03-09

**Authors:** Cheng-Da Wu, Te-Hua Fang, Fu-Yung Tung

**Affiliations:** 1 Department of Mechanical Engineering, Chung Yuan Christian University, 200, Chung Pei Rd., Chung Li District, Taoyuan 32023, Taiwan; nanowu@cycu.edu.tw; 2 Department of Mechanical Engineering, National Kaohsiung University of Applied Sciences, Kaohsiung 807, Taiwan; 1103303155@gm.kuas.edu.tw

**Keywords:** double-walled carbon nanotube, interface friction, friction coefficient, molecular dynamics

## Abstract

The interface friction characteristics of double-walled carbon nanotubes (DWCNTs) are studied using molecular dynamics simulations based on the Tersoff potential. The effects of the DWCNT type, outer shell diameter, and temperature are evaluated. The simulation results show that when an inner shell is being pulled out from a DWCNT, the friction force and normal force between shells increase with increasing the outer shell diameter. The noise of the friction force significantly increases with the increasing temperature. Zigzag@zigzag and armchair@armchair DWCNTs exhibit larger friction forces and smaller normal forces compared to those of chiral@chiral DWCNTs.

## 1. Introduction 

Carbon nanotubes (CNTs) are an important class of carbon-based materials due to their excellent physical properties, such as high mechanical strength, thermal conductivity, and electrical conductivity, low density, and large specific area [1]. Due to these remarkable properties, CNTs have many potential applications in micro-electromechanical systems (MEMS) [2], nano-electromechanical systems (NEMS) [3], and strain sensors [4], and as an adsorbent of flue gases [5]. In addition, the low interaction between adjacent shells of multiwalled carbon nanotubes (MWCNTs) due to van der Waals interactions has inspired studies on CNT-based oscillators and resonators.

Due to the difficulty of manipulation at the nano scale, there have been few experimental studies focusing on MWCNTs. Zhang et al. [6] investigated the interwall friction and sliding behavior of centimeter-long DWCNTs. They found that the interwall friction had a linear dependence on the pull-out velocity of an inner wall, and that the axial curvature of DWCNTs caused a significant increase in the interwall friction. The interwall friction has been found to be independent of the pull-out length [7]. Cumings et al. [8] and Akita et al. [9] observed that the pull-out force between nested walls in MWCNTs reaches a maximum, plateaus, and then drops suddenly.

Molecular dynamics (MD) simulation is a powerful tool for studying material interactions. Atomic simulation avoids experimental noise and turbulence problems, and can reduce cost. Many nanosystems have been successfully analyzed using MD, such as nanowelding [10,11], nanoforming [12], nanoextrusion [13], and nanotribology of metallic glasses [14]. Li et al. [15] studied the sliding behavior between nested walls in MWCNTs using MD simulations. They found that the pull-out force for MWCNTs is proportional to the diameter of the outer wall, and independent of nanotube length and chirality. Xia et al. [16] modeled a pull-out process for DWCNTs by applying a radial normal pressure on their outer wall and found that the pull-out force for fractured ends was much larger than that for capped ends. Song and Zha [17] studied the effect of intertube spacing on the sliding behavior of MWCNTs. Their study revealed that a small intertube spacing provides an effective channel for load transfer between tubes, regardless of tube length and wall number.

At present, the understanding of the interfacial tribology of DWCNTs with various geometric characteristics and at various environmental temperatures is still not very clear. Investigations into this are required for the development of nanoscale resonators and oscillators, particularly based on CNTs and potentially multilayer graphene. In this work, MD simulations of an inner shell being pulled out from a DWCNT are performed to analyze the interfacial friction between DWCNT shells. The effects of the DWCNT type, outer shell diameter, and temperature are studied in terms of atomic trajectories, friction force, and normal force.

## 2. Methodology

**Figure 1:**
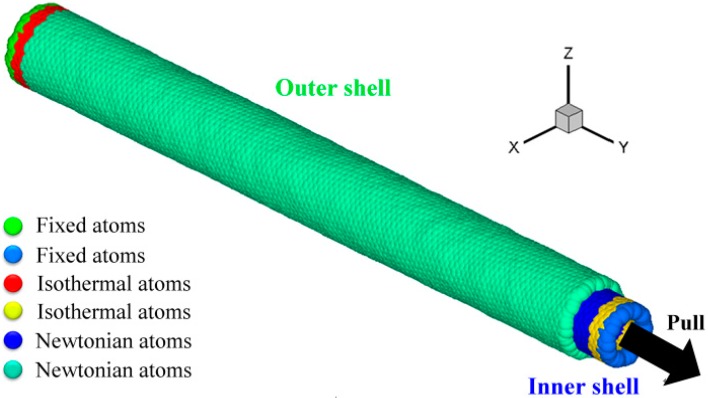
Molecular dynamics (MD) model of double-walled carbon nanotubes (DWCNTs). The model consists of an inner shell and an outer shell, whose lengths are both 30 nm. The diameters of the outer and inner shells are 2.73 and 2.4 nm, respectively.

Figure 1 shows an MD physical model of a DWCNT. The model consists of an inner shell and an outer shell, whose lengths are both 30 nm. Two layers of atoms on the left end of the outer shell were set as fixed layers to support the whole system. Two other layers of atoms next to the fixed layers were set as isothermal atoms, which were used to ensure that the heat generated during the pull-out process is dispersed outside the analytical region (layers of Newtonian atoms), keeping the temperature in the layers constant. The rest of the material was set as layers of Newtonian atoms. Newtonian atoms here mean atoms whose motion is described by Newton’s second law of motion without velocity scaling. For the inner shell, the order of layers was reversed (layers of fixed, isothermal, and Newtonian atoms were set from its right end). In order to analyze the interfacial friction between the inner and outer shells of DWCNTs during a relative motion process, the inner shell was moved by its fixed layers with a constant velocity of 40 m/s along the *Y*-direction. A total displacement of 27.5 nm was applied. No periodic boundary conditions were used in the model. The simulations are performed under a constant number of atoms, temperature, and volume (NTV ensemble).

The Tersoff-Brenner many-body potential function [18] was used to model the carbon-carbon atom interactions. This potential takes into account the coordination and angular dependence of the atoms and is well-suited to describing the intrashell covalent bonds. The long-range interactions of carbon were characterized using the Lennard-Jones (12-6) potential [19]. The cut-off radius, time step, and temperature in the simulation were set at 1.0 nm, 1 fs, and 300 K, respectively.

## 3. Results and Discussion

### 3.1. Effect of DWCNT Type

**Figure 2:**
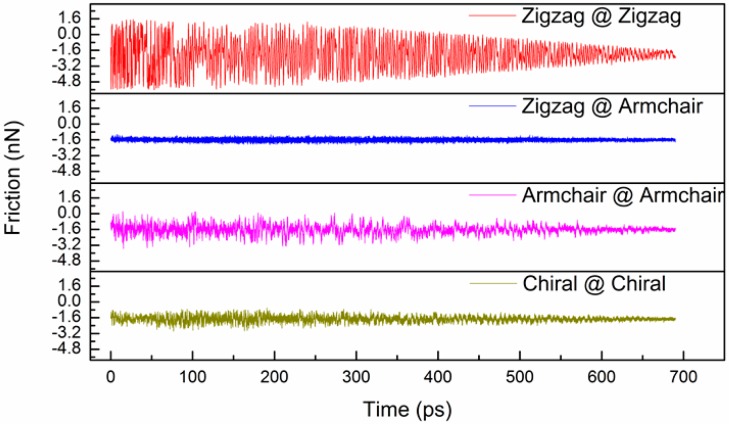
Variation of friction force between shells with time for four DWCNT types.

To study the effect of the DWCNT type, zigzag@zigzag (31,0)@(22,0), zigzag@armchair (31,0)@(12,12), armchair@armchair (18,18)@(12,12), and chiral@chiral (20,15)@(14,10) DWCNTs were used. These four types of DWCNT have approximate outer and inner shell diameters of 2.4 and 1.7 nm, respectively. The space between the shells is about 0.35 nm. Figure 2 shows the variation of the friction force between the shells with time for the four types of DWCNTs. The average friction force and normal force were evaluated by summing the lateral forces and normal forces that the inner shell atoms exerted on the outer shell atoms during the pull-out process, respectively. Both forces were averaged over a distance of 0.1 nm. The oscillation of the friction force curve decreased with the increasing time due to a decrease in the number of interacting atoms. The friction force curve oscillated with time due to a periodic stick-slip process [20]. The average friction forces were −2.3, −1.7, −1.8, and −1.9 nN for the zigzag@zigzag, zigzag@armchair, armchair@armchair, and chiral@chiral DWCNTs, respectively; the average normal forces were 0.032, 0.023, 0.019, and 0.058 nN, respectively. For zigzag@zigzag and armchair@armchair DWCNTs, the inner and outer shells meet the lattice matching requirement, i.e., there is commensurate contact between two graphene sheets [7,21]; therefore, they exhibit larger friction forces and smaller normal forces. The zigzag@armchair DWCNTs have the smallest average friction forces due to incommensurate contact. In other words, the two surfaces have no energetically preferred position with respect to one another, and so they can slide relative to each other with an extremely low energy cost [22]. The chiral@chiral DWCNTs thus have the largest normal forces due to incommensurate contact. With increasing the pull-out distance, the friction force curves in Figure 2 gradually converge to a fixed value of −1.6 nN. This is due to the effect of the chirality of CNTs on the friction force gradually decreasing with increasing the pull-out distance.

### 3.2. Effect of Outer Shell Diameter

**Figure 3:**
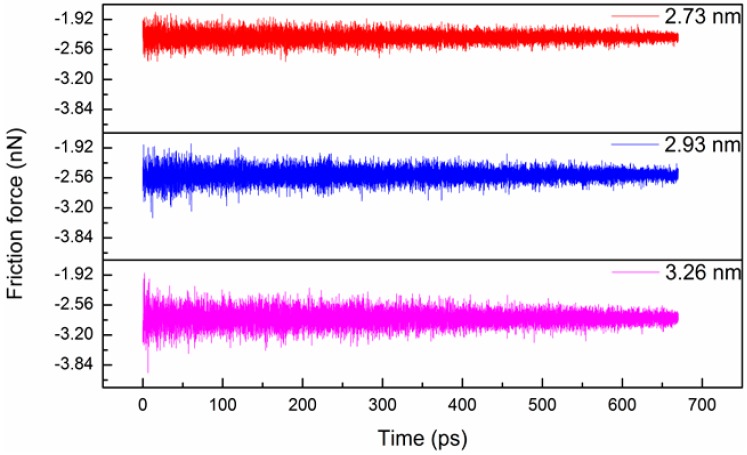
Variation of friction force between shells with time for three outer shell diameters.

**Figure 4:**
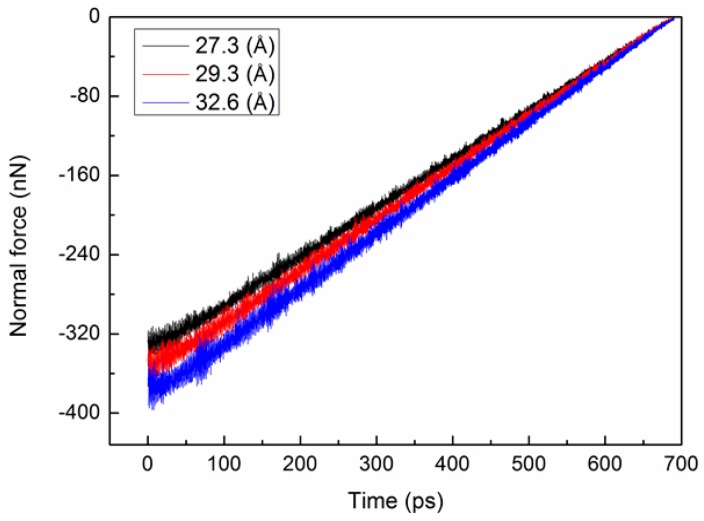
Variation of normal force between shells with time for three outer shell diameters.

**Figure 5:**
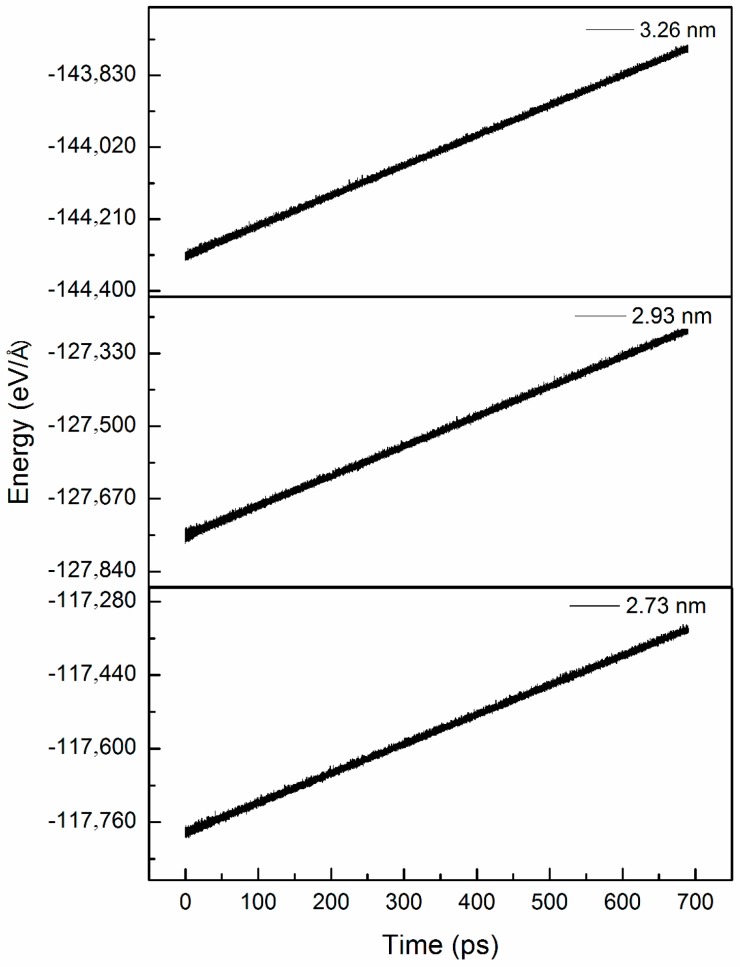
Variation of interaction energy with time for three outer shell diameters.

To study the effect of the outer shell diameter (*D*), three types of chiral@chiral DWCNTs, namely (22,18)@(14,10), (24,19)@(14,10), and (26,22)@(14,10), were used, corresponding to *D* values of 2.73, 2.93, and 3.26 nm, respectively. Figure 3 and Figure 4 show the variations of the friction force and normal force between the shells with time for the three *D* values. The average friction forces were −2.29, −2.49, and −2.84 nN for *D* values of 2.73, 2.93, and 3.26 nm, respectively. The friction force and normal force increased with the increasing *D* value due to an increase in the number of interacting atoms. The normal force reached its maximum at the beginning and then decreased with the increasing time. Figure 5 shows the variation of the interaction energy between the shells with time for the three *D* values. The interaction energy increased with the decreasing *D* value. 

### 3.3. Effect of Temperature

**Figure 6:**
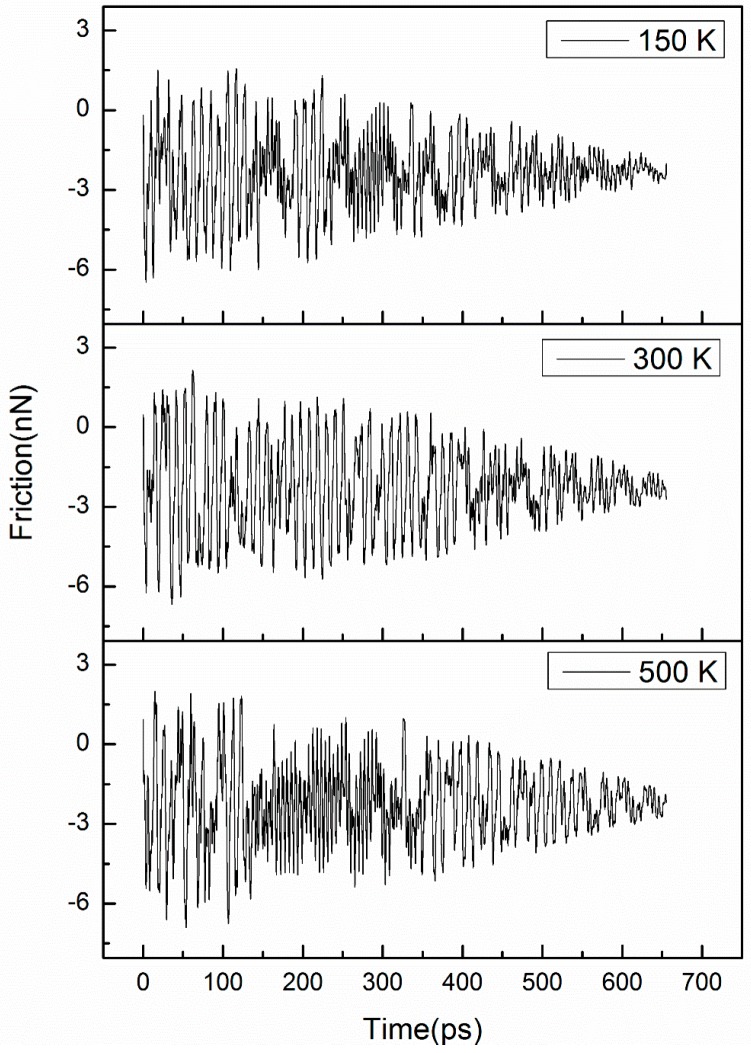
Variation of friction force between shells with time for temperatures of 150, 300, and 500 K.

**Figure 7:**
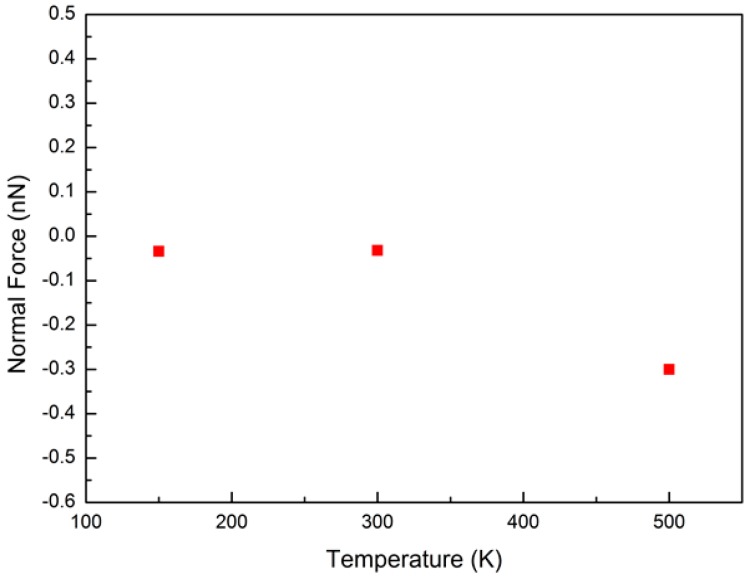
Variation of normal force between shells with time for temperatures of 150, 300, and 500 K.

To study the effect of temperature, three temperatures, namely 150, 300, and 500 K, were used. A zigzag@zigzag (31,0)@(22,0) DWCNT with inner and outer shell diameters of 1.75 and 2.47 nm, respectively, was used in the simulation. With the increasing temperature, the shell surfaces became more corrugated due to an increase in the kinetic energy of the atoms. The corrugation height is defined as the difference in the diameter of a given CNT. The corrugation height varied in the range of 0.24–0.27 nm for the outer shell surface when the temperature was increased from 150 to 500 K, and that for the inner shell was in the range of 0.1–0.16 nm. Figure 6 shows the variation of the friction force with time for the three temperatures. The noise of the friction force significantly increased with the increasing temperature due to an increase in the thermal fluctuation. For these tested temperatures, the average friction force was about −2.42 nN. This independence of friction on temperature disagrees with a previous report by Szlufarska et al., which showed that the friction force decreases with increasing temperature [23,24] because thermal excitations help overcome energy barriers and reduce the stick-slip jump magnitude. This could be concluded as an extremely low friction between shells. However, the average normal force increases with increasing temperature, as shown in Figure 7. 

## 4. Conclusions

MD simulations were used to investigate the effects of the DWCNT type, outer shell diameter, and temperature on the interfacial friction of DWCNTs. It was found that the friction force and normal force between shells increase with an increasing outer shell diameter and that the noise of the friction force between shells increases with an increasing temperature. Zigzag@zigzag and armchair@armchair DWCNTs exhibited larger friction forces and smaller normal forces than those of chiral@chiral DWCNTs.
